# Supplementary material for: Volatile scent chemicals in the urine of the red fox, Vulpes vulpes
Source: PLoS One. 2021 Mar 30;16(3):e0248961. doi: 10.1371/journal.pone.0248961 (PMC8009367; doi:10.1371/journal.pone.0248961)
Supplement: S1 Text — (DOCX) [file pone.0248961.s001.docx]

**S1 Text. References for Table 1**

1. McLean S, Davies NW, Nichols DS. Scent chemicals of the tail gland of the red fox, *Vulpes vulpes*. Chemical Senses. 2019;44(3):215-24. doi: 10.1093/chemse/bjz009.

2. Arnold J. Olfactory communication in red foxes (*Vulpes vulpes*) [PhD thesis]: University of Bristol; 2009.

3. Jorgenson JW, Novotny M, Carmack M, Copland GB, Wilson SR, Katona S, et al. Chemical scent constituents in the urine of the red fox (*Vulpes vulpes* L.) during the winter season. Science. 1978;199(4330):796-8. doi: 10.1126/science.199.4330.796.

4. Bailey S, Bunyan PJ, Page JMJ, editors. Variation in the levels of some components of the volatile fraction of urine from captive red foxes (*Vulpes vulpes*) and its relationships to the state of the animal. Chem Signals: Vertebr Aquat Invertebr; 1980 1980: Plenum, New York, N. Y; 1980.

5. Albone ES. Dihydroactinidiolide in supracaudal scent gland secretion of red fox. Nature. 1975;256(5518):575-. doi: 10.1038/256575a0.

6. Raymer J, Wiesler D, Novotny M, Asa C, Seal US, Mech LD. Chemical scent constituents in urine of wolf (*Canis-lupus*) and their dependence on reproductive hormones. Journal of Chemical Ecology. 1986;12(1):297-313. doi: 10.1007/bf01045612.

7. Osada K, Kurihara K, Izumi H, Kashiwayanagi M. Pyrazine analogues are active components of wolf urine that induce avoidance and freezing behaviours in mice. Plos One. 2013;8(4):e61753, 1-9. doi: e6175310.13 71/journal.pone.0061753.

8. Raymer J, Wiesler D, Novotny M, Asa C, Seal US, Mech LD. Chemical investigations of wolf (*canis, lupus*) anal-sac secretion in relation to breeding-season. Journal of Chemical Ecology. 1985;11(5):593-608. doi: 10.1007/bf00988570.

9. Martin J, Barja I, Lopez P. Chemical scent constituents in feces of wild Iberian wolves (*Canis lupus signatus*). Biochemical Systematics and Ecology. 2010;38(6):1096-102. doi: 10.1016/j.bse.2010.10.014.

10. Schultz TH, Flath RA, Stern DJ, Mon TR, Teranishi R, Kruse SM, et al. Coyote estrous urine volatiles. Journal of Chemical Ecology. 1988;14(2):701-12. doi: 10.1007/bf01013917.

11. Schultz TH, Kruse SM, Flath RA. Some volatile constituents of female dog urine. Journal of Chemical Ecology. 1985;11(Copyright (C) 2012 American Chemical Society (ACS). All Rights Reserved.):169-75. doi: 10.1007/bf00988199.

12. Natynczuk S, Bradshaw JWS, Macdonald DW. Chemical-constituents of the anal sacs of domestic dogs. Biochemical Systematics and Ecology. 1989;17(1):83-7. doi: 10.1016/0305-1978(89)90047-1.

13. Williams DJ. Tryptophan-metabolism and urinary quinoline bases in the greyhound. Research in Veterinary Science. 1986;41(2):273-4. doi: 10.1016/s0034-5288(18)30613-1.

14. Apps P, Mmualefe L, McNutt JW. Identification of volatiles from the secretions and excretions of African wild dogs (*Lycaon pictus*). Journal of Chemical Ecology. 2012;38(11):1450-61. doi: 10.1007/s10886-012-0206-7.

15. Osada K, Tashiro T, Mori K, Izumi H. The Identification of Attractive Volatiles in Aged Male Mouse Urine. Chemical Senses. 2008;33(9):815-23. doi: 10.1093/chemse/bjn045.

16. Ghoos Y, Claus D, Geypens B, Hiele M, Maes B, Rutgeerts P. Screening method for the determination of volatiles in biomedical samples by means of an off-line closed-loop trapping system and high-resolution gas chromatography-ion trap detection. Journal of Chromatography A. 1994;665(2):333-45. doi: 10.1016/0021-9673(94)85062-3.

17. Mochalski P, Unterkofler K. Quantification of selected volatile organic compounds in human urine by gas chromatography selective reagent ionization time of flight mass spectrometry (GC-SRI-TOF-MS) coupled with head-space solid-phase microextraction (HS-SPME). Analyst. 2016;141(15):4796-803. doi: 10.1039/c6an00825a.

18. Goodrich BS, Gambale S, Pennycuik PR, Redhead TD. Volatiles from feces of wild male house mice - chemistry and effects on behavior and heart-rate. Journal of Chemical Ecology. 1990;16(7):2091-106. doi: 10.1007/bf01026922.

19. Rock F, Mueller S, Weimar U, Rammensee HG, Overath P. Comparative analysis of volatile constituents from mice and their urine. Journal of Chemical Ecology. 2006;32(6):1333-46. doi: 10.1007/s10886-006-9091-2.

20. Goodwin TE, Rasmussen LEL, Schulte BA, Brown PA, Davis BL, Dill WM, et al. Chemical analysis of preovulatory female African elephant urine: A search for putative pheromones. In: Mason RT, LeMaster MP, MullerSchwarze D, editors. Chemical Signals in Vertebrates 10. 10 2005. p. 128-39.

21. Andersen KF, Vulpius T. Urinary volatile constituents of the lion, *Panthera leo*. Chemical Senses. 1999;24(2):179-89.

22. Sokolov VE, Albone ES, Flood PF, Heap PF, Kagan MZ, Vasilieva VS, et al. Secretion and secretory-tissues of the anal sac of the mink, *Mustela vison* - Chemical and histological studies. Journal of Chemical Ecology. 1980;6(4):805-25. doi: 10.1007/bf00990405.

23. Wanlong Z, Fangyan Y, Zhengkun W. Study of chemical communication based on urine in tree shrews *Tupaia belangeri* (Mammalia: *Scandentia: Tupaiidae*). European Zoological Journal. 2017;84(1):512-24. doi: 10.1080/24750263.2017.1391340.

24. Mills GA, Walker V. Headspace solid-phase microextraction profiling of volatile compounds in urine: application to metabolic investigations. Journal of Chromatography B-Analytical Technologies in the Biomedical and Life Sciences. 2001;753(2):259-68. doi: 10.1016/s0378-4347(00)00554-5.

25. Mattina MJI, Pignatello JJ, Swihart RK. Identification of volatile components of bobcat (*Lynx-rufus*) urine. Journal of Chemical Ecology. 1991;17(2):451-62. doi: 10.1007/bf00994344.

26. Miller KV, Jemiolo B, Gassett JW, Jelinek I, Wiesler D, Novotny M. Putative chemical signals from white-tailed deer (*Odocoileus virginianus*): Social and seasonal effects on urinary volatile excretion in males. Journal of Chemical Ecology. 1998;24(4):673-83. doi: 10.1023/a:1022342219469.

27. Ma WD, Wiesler D, Novotny MV. Urinary volatile profiles of the deermouse (Peromyscus maniculatus) pertaining to gender and age. Journal of Chemical Ecology. 1999;25(3):417-31. doi: 10.1023/a:1020937400480.

28. Soso SB, Koziel JA. Characterizing the scent and chemical composition of *Panthera leo* marking fluid using solid-phase microextraction and multidimensional gas chromatography-mass spectrometry-olfactometry. Scientific Reports. 2017;7:5137. doi: 10.1038/s41598-017-04973-2.

29. Chalmers RA, Healy MJR, Lawson AM, Watts RWE. Urinary organic-acids in man .2. Effects of individual variation and diet on urinary-excretion of acidic metabolites. Clinical Chemistry. 1976;22(8):1288-91.

30. Nielsen LT, Eaton DK, Wright DW, Schmidt-French B. Characteristic odors of *Tadarida brasiliensis mexicana Chiroptera : Molossidae*. Journal of Cave and Karst Studies. 2006;68(1):27-31.

31. Burger BV, Visser R, Moses A, Le Roux M. Elemental sulfur identified in urine of cheetah, *Acinonyx jubatus*. Journal of Chemical Ecology. 2006;32(6):1347-52. doi: 10.1007/s10886-006-9056-5.

32. Hubner B, Geibel K, Angerer J. Gas-chromatographic determination of propylene-glycol and diethylene glycol ethers in urine. Fresenius Journal of Analytical Chemistry. 1992;342(9):746-8. doi: 10.1007/bf00321870.

33. Lee JE, Lim HH, Shin HS. Simultaneous determination of 15 BTEX hydroxyl biomarkers in urine by headspace solid-phase microextraction gas chromatography-mass spectrometry. Journal of Pharmaceutical and Biomedical Analysis. 2019;174:115-22. doi: 10.1016/j.jpba.2019.05.033.

34. Burger BV, Leroux M, Spies HSC, Truter V, Bigalke RC. Mammalian Pheromone Studies .4. Terpenoid Compounds and Hydroxy Esters from the Dorsal Gland of the Springbok, Antidorcas-Marsupialis. Zeitschrift Fur Naturforschung C-Journal of Biosciences. 1981;36(3-4):340-3.

35. Andersen KK, Bernstein DT, Caret RL, Romanczyk LJ. Chemical-constituents of the defensive secretion of the striped skunk (*Mephitis-mephitis*). Tetrahedron. 1982;38(13):1965-70. doi: 10.1016/0040-4020(82)80046-x.

36. Rendon NM, Soini HA, Scotti MAL, Novotny MV, Demas GE. Urinary volatile compounds differ across reproductive phenotypes and following aggression in male Siberian hamsters. Physiology & Behavior. 2016;164:58-67. doi: 10.1016/j.physbeh.2016.05.034.

37. de la Pena E, Martin J, Carranza J. The intensity of male-male competition may affect chemical scent constituents in the dark ventral patch of male Iberian red deer. Plos One. 2019;14(9):e0221980. doi: 10.1371/journal.pone.0221980.

38. Setchell JM, Vaglio S, Moggi-Cecchi J, Boscaro F, Calamai L, Knapp LA. Chemical composition of scent-gland secretions in an old world monkey (*Mandrillus sphinx*): Influence of sex, male status, and individual identity. Chemical Senses. 2010;35(3):205-20. doi: 10.1093/chemse/bjp105.

39. Wood WF, Sollers BG, Dragoo GA, Dragoo JW. Volatile components in defensive spray of the hooded skunk, *Mephitis macroura*. Journal of Chemical Ecology. 2002;28(9):1865-70. doi: 10.1023/a:1020573404341.

40. Vogt K, Boos S, Breitenmoser U, Kolliker M. Chemical composition of Eurasian lynx urine conveys information on reproductive state, individual identity, and urine age. Chemoecology. 2016;26(6):205-17. doi: 10.1007/s00049-016-0220-2.

41. Burger BV, Viviers MZ, Bekker JPI, le Roux M, Fish N, Fourie WB, et al. Chemical characterization of territorial marking fluid of male Bengal tiger, *Panthera tigris*. Journal of Chemical Ecology. 2008;34(5):659-71. doi: 10.1007/s10886-008-9462-y.

42. Zhang JX, Soini HA, Bruce KE, Wiesler D, Woodley SK, Baum MJ, et al. Putative chemosignals of the ferret (*Mustela furo*) associated with individual and gender recognition. Chemical Senses. 2005;30(9):727-37. doi: 10.1093/chemse/bji065.

43. Ninomiya K, Nohara I, Toyoda T, Kimura T. The pattern of volatile compounds in incubated and fresh preputial fluid of male-mice. Zoological Science. 1993;10(3):537-42.

44. Cheng M, et al. GC-MS analysis on volatile components of wild Trogopterus feces from Shanyang county of Shaanxi province. Zhongguo Shiyan Fangjixue Zazhi. 2011;17(19):97-100.

45. Arnaiz E, Moreno D, Quesada R. Determination of volatiles in mouse urine by headspace solid phase microextraction and gas chromatography-mass spectrometry. Analytical Letters. 2014;47(5):721-9. doi: 10.1080/00032719.2013.853182.

46. McLean S, Davies NW, Wiggins NL. Scent chemicals of the brushtail possum, *Trichosurus vulpecula*. Journal of Chemical Ecology. 2012;38(10):1318-39. doi: 10.1007/s10886-012-0188-5.

47. Dzieciol M, Woszczylo M, Szumny A, Jezierski T, Kupczynski R, Godzinska EJ, et al. Identification of putative volatile sex pheromones in female domestic dogs (*Canis familiaris*). Animal Reproduction Science. 2018;197:87-92. doi: 10.1016/j.anireprosci.2018.08.016.
