# Supplementary material for: Volatile scent chemicals in the urine of the red fox, Vulpes vulpes
Source: PLoS One. 2021 Mar 30;16(3):e0248961. doi: 10.1371/journal.pone.0248961 (PMC8009367; doi:10.1371/journal.pone.0248961)
Supplement: S2 Text — (DOCX) [file pone.0248961.s002.docx]

**S2 Text. Identification of compounds without a reference KI Value**

Seven compounds were identified without a reference standard or published Kovats retention index. In each case the mass spectrum matched the NIST reference data [1]. Three are unknowns whose structure could not be determined (compounds 53, 59 and 63).

15. 3-Isopentenyl thiol (15) KI 794


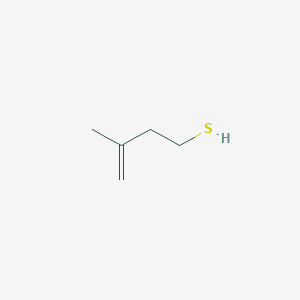
C_5_H_10_S MW 102

The mass spectrum (MS) matched the NIST MS, and showed ions which are characteristic of this structure. Found *m/z* 102.2 M^+^ (100%), 104.1 [^34^S isotope ion] 2.6%, 87.1 [M-15]^+^, 22%, 47.1 [CH_2_SH]^+^ 40%. Note that the ^34^S isotope ion usually has an abundance about 4% of the corresponding ^32^S ion, for each sulfur atom in the molecule [2]. Supporting evidence is given by presence S-methyl homologue (compound no. 20) which eluted later (KI 896). The isopentyl/isopentenyl structure is found is four compounds (compounds 12, 15, 17, and 20).

35. Nonanal, branched (35) KI 1087

C_9_H_11_O MW 142

This compound had the same MS as *n*-nonanal (compound 37, KI 1101) but eluted 14 KI units earlier, indicating a branched carbon chain. A literature search found a KI 1058 for *anteiso*-nonanal (6-methyl octanal), demonstrating the effect of branching at this position. It is not possible to identify the position of the branch in compound 35.

46. 1-Phenylethyl methyl sulfide (46) KI 1196


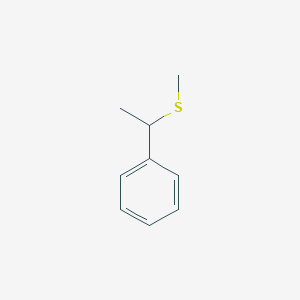
C_9_H_12_S MW 152

The MS showed characteristic ions at *m/z* 152.2 [M]^+^ (24%), 154.3 [^34^S isotope of M+] (5.5% of the abundance of ^32^S M^+^), 105.1 [M – SCH_3_]^+^ 100%. Also present was the corresponding thiol (38) at KI 1121.

51. 2-Phenylethyl methyl sulfide (51) KI 1280


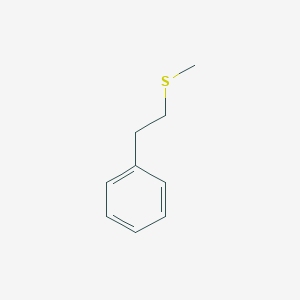
 C_9_H_12_S MW 152

This compound is an isomer of compound 46 and its MS shows the same M^+^ ion and different fragmentations which are characteristic of the unbranched sidechain. Ions at *m/z* 152.2 [M]^+^ 88%, 154.4 [^34^S M^+^] 5.6% of ^32^S M^+^), 104.2 [M - (SCH_3_ + H)]^+^ 100%), 91.2 [M – (CH_2_SCH_3_)]^+^ 66%, 61.1 [CH_2_SCH_3_]^+^ 93%. Also present was the corresponding thiol (44) at KI 1174.

57. Decanoic acid, branched (57) KI 1348

C_10_H_20_O_2_ MW 172

This compound has the same MS as *n*-decanoic acid, which eluted at KI 1373, 25 units later. Branching causes fatty acids and other alkanes to elute earlier. This was not further investigated, but we have previously shown that by GC-MS analysis of the methyl esters of fatty acids the KI offsets from the value for the straight chain *n*-acids can in many cases enable the position of the methyl group to be assigned [3].

60. 2-Methylquinoline, 8-hydroxy- (60)


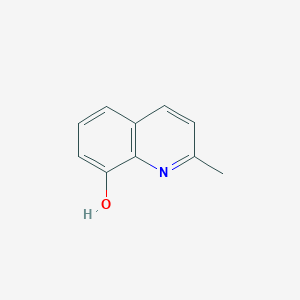
C_10_H_9_O MW 159

This compound could be formed metabolically from 2-methylquinoline (compound 55, KI 1309). Its MS matched the NIST reference data. Ions at 159.2 M^+^ (100%), 131.3 (77%), 130 (33%), 129 (1.1%), 128 (7.8%).

The 2-hydroxymethyl derivative (CAS 1780-17-2) is also a possible metabolite of 2-methylquinoline. Williams [4] showed that radiolabelled tryptophan was excreted in urine as 2-aminomethylquinoline (CAS 5760-20-3) that, as urine aged, degraded successively to 2-aminoquinoline and 2-hydroxymethylquinoline. However, its mass spectrum (from NIST) shows a set of ions at *m/z* 128-131 which do not match the mass spectrum of compound 60.

Another compound (which is unrelated to 2-methylquinoline) with a mass spectrum similar to that of compound 60 is 8-hydroxymethylquinolone (CAS 16032-35-2). However, it seems far less likely as it is comparatively rare with only 40 references in SciFinder compared to 1633 references to 8-hydroxy-2-methylquinoline. This compound has many uses including as an antimicrobial and has been found in landfill leachates [5] and is therefore considered likely to be compound 60.

**References**

1. NIST. NIST Mass Spectral Library Version 2.2. National Institute of Standards and Technology. USA 2014

2. Beynon JH, Saunders RA, Williams AE. The Mass Spectra of Organic Molecules. Amsterdam: Elsevier; 1968. 510 p.

3. McLean S, Davies NW, Nichols DS. Lipids of the tail gland, body and muzzle fur of the red fox, *Vulpes vulpes*. Lipids. 2017;52(7):599-617. doi: 10.1007/s11745-017-4270-1.

4. Williams DJ. Tryptophan-metabolism and urinary quinoline bases in the greyhound. Research in Veterinary Science. 1986;41(2):273-4. doi: 10.1016/s0034-5288(18)30613-1.

5. Yang ZQ, Zhou SQ. The biological treatment of landfill leachate using a simultaneous aerobic and anaerobic (SAA) bio-reactor system. Chemosphere. 2008;72(11):1751-6. doi: 10.1016/j.chemosphere.2008.04.090.
