# Supplementary figures and images for: Volatile scent chemicals in the urine of the red fox, Vulpes vulpes
Source: PLoS One. 2021 Mar 30;16(3):e0248961. doi: 10.1371/journal.pone.0248961 (PMC8009367; doi:10.1371/journal.pone.0248961)

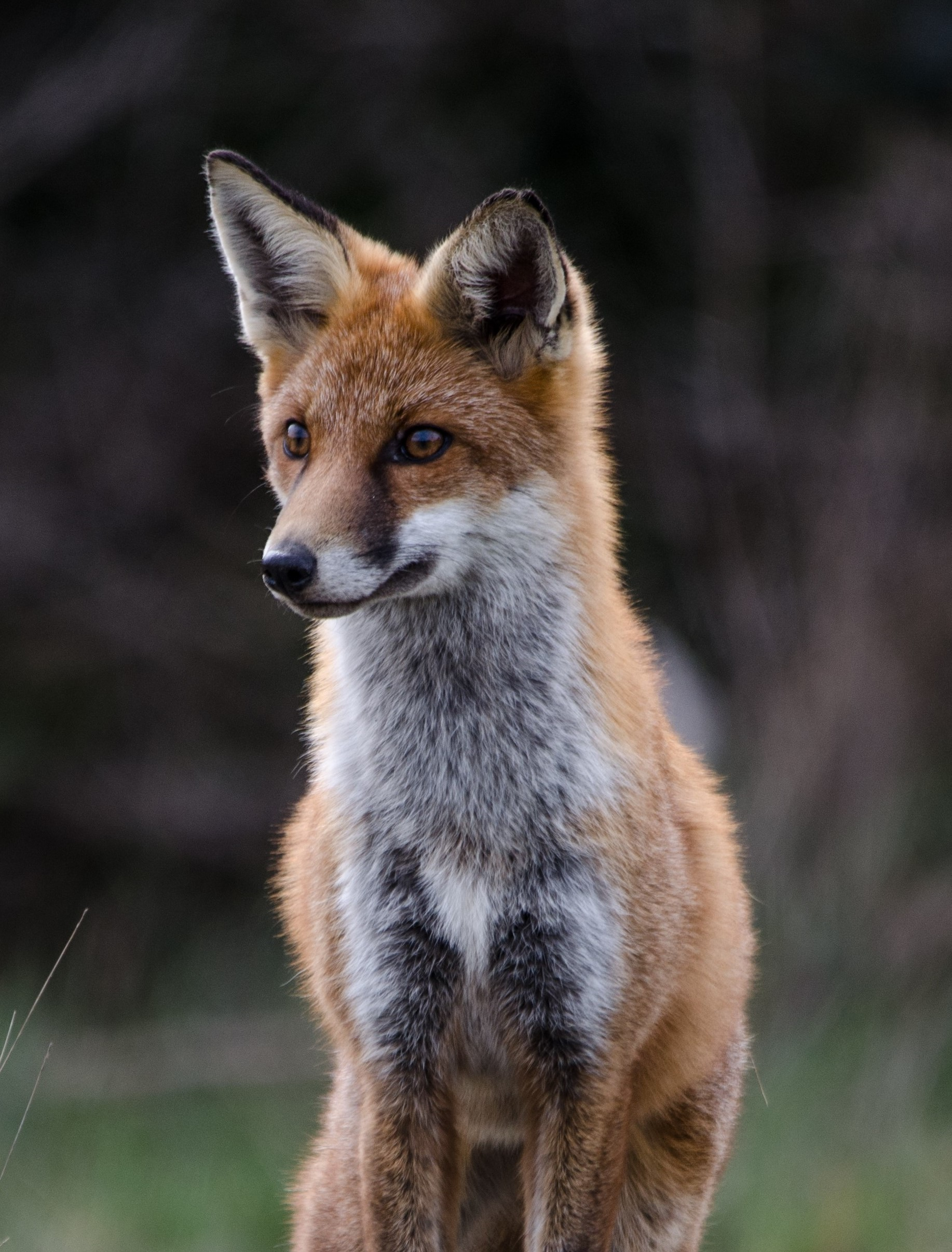

Supplement: S1 Fig — (TIF) [file pone.0248961.s003.tif]
