# Supplementary material for: Volatile scent chemicals in the urine of the red fox, Vulpes vulpes
Source: PLoS One. 2021 Mar 30;16(3):e0248961. doi: 10.1371/journal.pone.0248961 (PMC8009367; doi:10.1371/journal.pone.0248961)
Supplement: Attachment — Submitted filename: comment.doc [file pone.0248961.s004.doc]

In this manuscript entitled “Volatile scent chemicals in the urine of the red fox, *Vulpes vulpes*” Stuart Mc Lean *et. al.* studied the chemical composition of fox urine using solid-phase microextraction and gas chromatography-mass spectrometry to analyze the different urinary volatiles in a number of free-ranging wild foxes living in farmlands and bushes in Victoria, Australia. Compounds were identified from their mass spectra and Kovats retention indices. Identified compounds include endogenous scent compounds, various plant derived compounds and anthropogenic xenobiotics. These urinary odorant compounds may represent a highly evolved system of semiochemicals for communication.

The results depicted are well presented, correlated to each other and properly explained (except in some aspect) providing a deep insight into the semiochemical interaction. The work depits a very interesting behavioural aspect of the species, thus apt for the theme of this journal and the manuscript may be accepted for publication once these points are addressed:

1. The results demand the raw data of the gas chromatography-mass spectrometry analysis, may be presented as supplimentary file.
2. The percentage of the compounds should be represented by statistical analysis with ± values.
3. In the text the authors mentioned the urinary volatiles from both male and female species. How the composition of the chemicals varied in male and female species? Was there any major differences in the composition? What is the significance of such variations in the volatile composition? It will be appreciated if the authors highlight these aspects with proper explanation.
4. Minor errors: Take care of the few typographical errors in the text.
