# Supplementary material for: Volatile scent chemicals in the urine of the red fox, Vulpes vulpes
Source: PLoS One. 2021 Mar 30;16(3):e0248961. doi: 10.1371/journal.pone.0248961 (PMC8009367; doi:10.1371/journal.pone.0248961)
Supplement: Attachment — Submitted filename: Response to Reviewers.docx [file pone.0248961.s005.docx]

PONE-D-20-33017
Volatile Scent Chemicals in the Urine of the Red Fox, Vulpes vulpes
PLOS ONE

**The authors comments are inserted in blue text. The page and line references are to the Revised Manuscript with Track Changes. Inserted text is enclosed by quotation marks.**

**Comments to the Author**

1. Is the manuscript technically sound, and do the data support the conclusions?

The manuscript must describe a technically sound piece of scientific research with data that supports the conclusions. Experiments must have been conducted rigorously, with appropriate controls, replication, and sample sizes. The conclusions must be drawn appropriately based on the data presented.

Reviewer #1: Yes

Reviewer #2: Partly

2. Has the statistical analysis been performed appropriately and rigorously?

Reviewer #1: Yes

Reviewer #2: No

3. Have the authors made all data underlying the findings in their manuscript fully available?

The [PLOS Data policy](https://protect-au.mimecast.com/s/MGGcCp8AG6fBrWYLi79L_M?domain=plosone.org) requires authors to make all data underlying the findings described in their manuscript fully available without restriction, with rare exception (please refer to the Data Availability Statement in the manuscript PDF file). The data should be provided as part of the manuscript or its supporting information, or deposited to a public repository. For example, in addition to summary statistics, the data points behind means, medians and variance measures should be available. If there are restrictions on publicly sharing data—e.g. participant privacy or use of data from a third party—those must be specified.

Reviewer #1: Yes

Reviewer #2: No

Data will be held in a public repository at the University of Tasmania.

<https://rdp.utas.edu.au> The DOI will be available on acceptance of this manuscript.

4. Is the manuscript presented in an intelligible fashion and written in standard English?

PLOS ONE does not copyedit accepted manuscripts, so the language in submitted articles must be clear, correct, and unambiguous. Any typographical or grammatical errors should be corrected at revision, so please note any specific errors here.

Reviewer #1: Yes

Reviewer #2: Yes

5. Review Comments to the Author

Please use the space provided to explain your answers to the questions above. You may also include additional comments for the author, including concerns about dual publication, research ethics, or publication ethics. (Please upload your review as an attachment if it exceeds 20,000 characters)

Reviewer #1: Strengths

One strength of this work is it has revealed a greater number of compounds in the urine of red foxes than previous studies. As mentioned by the authors, the urine of red foxes was first analyzed four decades prior to this work by Jorgenson et al. [1] who used gas chromatography with a nitrogen and sulfur selective detectors, which only allowed for the identification of eight compounds. In contrast, the authors of this work used gas chromatography-mass spectrometry (a universal detector), which has allowed them to detect and presumptively identify 69 compounds from red foxes. By revealing a larger array of compounds, scientists can now begin to explore their role in chemical signaling among conspecifics.

Another strength to this work is the sampling methods. By removing urine directly from the bladders of culled red foxes (often within 30 minutes of death), they were able to retrieve a native sample that is free of any external materials. This ensured that their results were unambiguous.

Lastly, I appreciated how the authors tried to accurately identify the compounds. It is common practice to use NIST library matches to get a tentative idea of which compounds are present in samples, but I believe it is essential to use chemical standards to confirm their identity. However, I understand it can be difficult to obtain a standard for each compound when working with upwards of 50 compounds. I was impressed to see they were able to purchase standards for 41 compounds and then use Kovat’s retention index to confirm the rest. These combined efforts allowed them to positively identify a total 59 compounds.

Weaknesses

The data was a bit overwhelming to analyze and isn’t user friendly. The primary source of data was presented in Table 1, which was chock-full of information thus making it hard to process. In my opinion, it would be best to take the information in Table 1 and distribute it into different tables and graphs, each with their own objective. For instance, a bar graph could compare the number of foxes a compound was identified from. Another figure could include box and whisker plots of the percentage of total compounds quantitated for each compound. Lastly, a table could be used to compare which compounds were identified in other species.

The quantitative data have been removed from Table 1 (pages 12-21) and placed in two new figures: **Fig 2. Number of foxes in which each compound was found.** **Fig 3. The relative abundance (%total) of each compound in all foxes** (Page 22) The relative percentages of each compound, summarised as quartiles and range. The compounds have now been numbered in Table 1, and the numbers are used in the new Figs and in the text. The numbers of the peaks in the chromatogram (Figure 1) have been changed to those now used in Table 1.

Table 1 (pages 17-21) now lists the compounds identified and their reported findings in foxes and other species. The references are still listed in a separate bibliography placed in S1 Text (as described below).

Additionally, I was disappointed to not see information specifically pertaining to the foxes sampled. It would have been interesting to see which compounds and the amounts of each compound (according to peak area) was collected from each fox in a stacked column graph.

These data have now been graphed in a new **Fig 4. The relative abundance (% total) of each compound found in individual foxes**. (page 23) This Fig shows the generally great abundance of acetophenone in fox urine, and this has prompted us to add a new paragraph in the Discussion, commenting on this finding (page 31).

On occasion, I found some of the descriptions hard to understand. The language was unambiguous, but the ideas were not presented in a clear and logical way. For example, the paragraph on page 6, lines 124-129 (under the section titled “Analysis by gas chromatography-mass spectrometry”) was not clear to me (see my comments in minor issues). I felt the same way about the “Quantitation and Statistical Analysis” section. I think it is safe to say that quantifying compounds was not the main objective of this paper; however, an attempt was made to quantify acetophenone and the sulfur compounds and I found these methods to be unconventional. There were a few more instances of confusing text in the results and discussion sections, as well.

An additional explanatory section has been added in the beginning of the Methods section (pages 5-6). “**Overview of analyses (etc)” – see in manuscript.**

Minor Issues

• Analysis by gas chromatography-mass spectrometry, lines 108-116: It is unclear when the deuterated acetophenone and the alkane mixture was added to the urine sample. These additives are mentioned after compound extraction. Please clarify.

This has been clarified in the text, in the paragraph: “Urine samples were thawed…”(page 7, lines 143-147).

• Analysis by gas chromatography-mass spectrometry, lines 124-129: This paragraph is confusing. Is this saying that some samples were analyzed using automated injection (robotic sampler) and the rest were analyzed using manual injection? Also, what was meant by the statement acetophenone was quantitated after samples were analyzed (line 124-125)? Please rewrite in a clear manner.

Replacement paragraph (together with the **Overview of analyses** section above):

(page 8, lines 163-168)

“Samples were initially analyzed within 4 months of collection, and the acetophenone was quantitated subsequently. Some samples had been collected before the automated apparatus was available (foxes 1-8), and their initial analyses used manual extraction and injection methods previously described [1]. For all samples, acetophenone was quantitated using the automated method as described in Overview of analyses.”

• Quantitation and Statistical Analysis: The word ‘quantitation’ is a bit misleading since mass amounts were not calculated and should probably be referred to as ‘compound abundance’ instead. Consider revising.

The text has been revised as suggested (at several sites).

• Quantitation and Statistical Analysis: In terms of acetophenone, the methods do not clearly state how it was quantified. If the concentration of acetophenone was calculated by comparison to deuterated acetophenone, then it is important that a response factor be established between the two compounds. Add text that explains how acetophenone was quantified.

Line 171, new paragraph (page 10):

“The GC-MS chromatograms showed that the TIC peaks for acetophenone and its d8-isomer did not completely resolve. Their abundances were measured by ion plots of *m/z* 120 + 105 + 77 for acetophenone (comprising 0.8023 TIC) and *m/z* 128 + 110 + 82 for d_8_-acetophenone (0.783 TIC). The response factor acetophenone/d_8_-isomer was 0.962. “

This response factor has now been applied to the calculations of acetophenone concentrations, making a small change in the values (they are reduced by the ration 0.962). The values have been changed for the concentrations of acetophenone in μg/ml (pages 2 and 23) and sulfur compounds in μg ‘acetophenone equivalents’/ml (page 25)

• Figure 1: Is deuterated acetophenone included in this sample? If so, I think it would be best to indicate its peak.

No, this figure shows a qualitative GC-MS analysis without added d_8_-acetophenone or alkane standards.

• Table 1: As I mentioned previously, I think it would be best to distribute the information in Table 1 into multiple different tables and graphs, each with their own objective. For instance, a bar graph could compare the number of foxes (y-axis) a compound was identified from (x-axis). Another figure could include box and whisker plots of the percentage of total compounds quantitated for each compound. Lastly, a table could be used to compare which compounds were identified in other species.

This has been done, as explained above (Figs 2-4).

• Table 1, RI or KI lit: Some compounds have a dash listed. How were their identities confirmed?

These compounds were identified from their mass spectra and KI data, as now described in **supporting information S2.** In doing this, some other corrections were made in Table 1. The CAS no. for β-myrcene was corrected, and a literature value KI for cpd 58 Texanol added

(KI value from Reidel RVB, Cioni PL, Pistelli L. Volatile emission of different plant parts and fruit development from Italian cherry plums (*Prunus cerasifera* and *P. cerasifera 'Pissrdii*'). Biochemical Systematics and Ecology. 2017;75:10-7. doi: 10.1016/j.bse.2017.10.001.)

The hydroxy position in 2-methylquinoline (compound 60) is 8-, not 2-, as now discussed in **S2. Identification of compounds without a reference KI value**.

A recent new reference on the finding of acetophenone in dog urine has been added to Table 1. (Dzieciol M, Woszczylo M, Szumny A, Jezierski T, Kupczynski R, Godzinska EJ, et al. Identification of putative volatile sex pheromones in female domestic dogs (Canis familiaris). Animal Reproduction Science. 2018;197:87-92. doi: 10.1016/j.anireprosci.2018.08.016.)

• Table 1, Reports in canids: The references do not appear to be correct. For example, with regards to ethyl acetate, T1 is listed under fox. I interpret that as ethyl acetate was detected in the tail gland of a fox as it appears in reference #1, but reference number 1 by Saunders et al. [2] does not mention ethyl acetate anywhere in their review. Update references in the table to match the correct citation.

The references giving reports on other animals are compiled in a separate bibliography which is placed in the supporting information **S1 File. References for Table 1**. This is to avoid expected formatting difficulties in using the Endnote software with the Microsoft Word Table, and because these references would considerably expand the main bibliography and that it was considered that few readers would wish to consult them.

• Table 1, Human HMDB: Under beta-myrcene it lists, ‘check’. Is that supposed to be there?

No; thanks. Now corrected. Also the CAS number. (page 18, compound no. 27)

• Table 1, Human HMDB: An X indicates that the compound was not reported in the database. I would suggest this be removed to be consistent with the other species. This table is already full of information, it might be best to remove unnecessary symbols.

OK, the Xs have now been removed (pages 17-21).

• Table 1, Human HMBD: The symbols are confusing. Only N and X have been described, but symbol such as U and F are included. Does U mean urine and F mean feces as seen in the other columns?

Yes, now clarified in the Table footnotes (page 21)

• Results, Line 304: It is mentioned that 2-aminoacetophenone was previously found in ferret urine and feces, but no reference is provided. Add reference.

Added Zhang 2005 (ref 41) (page 26, line 390)

• Results, Line 310: The sentence begins by saying six compounds of human origin were present in fox urine, which includes deuterated acetophenone; however, wasn’t deuterated acetophenone added to each sample? Consider revising this sentence.

Yes, thanks. It now reads “five compounds” page 26, lines 401)

References

(cut here)

Reviewer #2: In this manuscript entitled “Volatile scent chemicals in the urine of the red fox, Vulpes vulpes” Stuart Mc Lean et. al. studied the chemical composition of fox urine using solid-phase microextraction and gas chromatography-mass spectrometry to analyze the different urinary volatiles in a number of free-ranging wild foxes living in farmlands and bushes in Victoria, Australia. Compounds were identified from their mass spectra and Kovats retention indices. Identified compounds include endogenous scent compounds, various plant derived compounds and anthropogenic xenobiotics. These urinary odorant compounds may represent a highly evolved system of semiochemicals for communication.
The results depicted are well presented, correlated to each other and properly explained (except in some aspect) providing a deep insight into the semiochemical interaction. The work depits a very interesting behavioural aspect of the species, thus apt for the theme of this journal and the manuscript may be accepted for publication once these points are addressed:
1.The results demand the raw data of the gas chromatography-mass spectrometry analysis, may be presented as supplimentary file.

The raw data are in Varian Workstation software which is not open source. The peak area data used for the TIC chromatogram will be available in the data repository as an Excel file. The mass spectral data are not readily made available as they require the Varian software.

2.The percentage of the compounds should be represented by statistical analysis with ± values.

Compared to the mean, the median was considered to be a more appropriate measure of the central value of the data since it is less affected by non-normal data which is skewed and contains outliers. Following the suggestion by Reviewer #1, the data have now been presented in a box and whisker graph of median, quartiles and range (Fig 3, page 22).

[3.In](https://protect-au.mimecast.com/s/eHOZCq7BJXtoA3qrCviybM?domain=3.in) the text the authors mentioned the urinary volatiles from both male and female species. How the composition of the chemicals varied in male and female species? Was there any major differences in the composition? What is the significance of such variations in the volatile composition? It will be appreciated if the authors highlight these aspects with proper explanation.

Regrettably, there were only two females, which was considered insufficient for making comparisons between the sexes.

4.Minor errors: Take care of the few typographical errors in the text.

All errors which have been detected have been corrected.

**Other changes, not requested by the reviewers**

Page 31 A new paragraph, commenting on the possible significance of acetophenone (as mentioned above, following the mention of the new Fig 4.

Page 34 A new reference on compound 60 has been added, and discussion of its identification moved to S2.
